# THE ROLE OF CONVENTIONAL ECHOENDOSCOPY (EUS) IN THERAPEUTIC DECISIONS IN PATIENTS WITH NEUROENDOCRINE GASTROINTESTINAL TUMORS

**DOI:** 10.1590/0102-672020190001e1512

**Published:** 2020-08-24

**Authors:** Rodrigo Dias da COSTA, Rafael KEMP, José Sebastião dos SANTOS, Débora Azeredo Pacheco Dias COSTA, José Celso ARDENGH, Jurandir Marcondes RIBAS-FILHO, Carmen Australia Paredes Marcondes RIBAS

**Affiliations:** 1 Postgraduate Program in Principles of Surgery, Evangelical Faculty of Paraná/University Evangelical Hospital of Curitiba/Medical Research Institute, Curitiba, PR, Brazil; 2 Digestive Endoscopy Service, 9 de Julho Hospital, São Paulo, SP, Brazil; 3 Section of Endoscopy, Hospital das Clínicas, Faculty of Medicine of Ribeirão Preto, University of São Paulo, Ribeirão Preto, SP, Brazil; 4 Postgraduate Program, Department of Diagnostic Imaging, Paulista School of Medicine, Federal University of São Paulo, São Paulo, SP, Brazil

**Keywords:** Endosonography, Carcinoid Tumor, Carcinoma, Neuroendocrine, Endossonografia, Tumor carcinoide, Carcinoma Neuroendócrino

## Abstract

**Background:**

Gastrointestinal neuroendocrine tumors are rare, usually presented as subepithelial or polypoid tumors. Accurate diagnosis and indication of the type of resection are still challenging**.**

**Aim:**

To determine the effectiveness of echoendoscopy in determining the depth of the lesions (T) identified by endoscopy in order to evaluate surgical and/or endoscopic indication, and to evaluate the results of endoscopic removal in the medium term.

**Methods:**

Twenty-seven patients were included, all of whom underwent echoendoscopy for TN tumor staging and the evaluation of possible endoscopic resection. The parameters were: lesion size, origin layer, depth of involvement and identified perilesional adenopathies. The inclusion criteria for endoscopic resection were: 1) high surgical risk; 2) those with NET <2 cm; 3) absence of impairment of the muscle itself; and 4) absence of perilesional adenopathies in echoendoscopy and in others without distant metastases. Exclusion criteria were TNE> 2 cm; those with infiltration of the muscle itself; with perilesional adenopathies and distant metastases. The techniques used were: resection with polypectomy loop; mucosectomy with saline injection; and mucosectomy after ligation with an elastic band. The anatomopathological study of the specimens included evaluation of the margins and immunohistochemistry (chromogranin, synaptophysin and Ki 67) to characterize the tumor. Follow-up was done at 1, 6 and 12 months.

**Results:**

Resections with polypectomy loop were performed in 15 patients; mucosectomy in five; mucosectomy and ligation with elastic band in three and the remaining four were referred for surgery. The anatomopathological specimens and immunohistochemical analyzes showed positive chromogranin and synaptophysin, while Ki 67 was less than 5% among all cases. The medium-term follow-up revealed three recurrences. The average size of tumors in the stomach was 7.6 mm and in the duodenum 7.2 mm. Well-demarcated, hypoechoic, homogeneous lesions occurred in 75%; mucous layer in 80%; and the deep and submucosal mucosa in 70%.

**Conclusions:**

Echoendoscopy proved to be a good method for the study of subepithelial lesions, being able to identify the layer affected by the neoplasm, degree of invasion, echogenicity, heterogeneity, size of the lesion and perilesional lymph node involvement and better indicate the treatment option.

## Graphical Abstract

[Fig f1a]

**Figure f1a:**
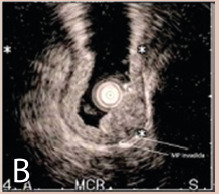
Gastric neuroendocrine tumor showing invasion of the muscle layer?

## RESUMO

**Racional:**

Tumores neuroendócrinos gastrointestinais são raros geralmente apresentados como tumores subepiteliais ou polipoides. O diagnóstico preciso e a indicação do tipo de ressecção ainda são desafiadores.

**Objetivo:**

Determinar a eficácia da ecoendoscopia em determinar a profundidade das lesões (T) identificadas pela endoscopia com objetivo de avaliar indicação cirúrgica e/ou endoscópica, e avaliar os resultados da remoção endoscópica em seguimento em médio prazo.

**Métodos:**

Foram incluídos 27 pacientes todos submetidos à ecoendoscopia para estadiamento tumoral TN e à avaliação de possível ressecção endoscópica. Os parâmetros estudados foram: tamanho da lesão, camada de origem, profundidade do acometimento e adenopatias perilesionais identificadas. Os critérios de inclusão para ressecção endoscópica foram: 1) risco cirúrgico elevado; 2) aqueles com TNE <2 cm; 3) ausência de comprometimento da muscular própria; e 4) ausência de adenopatias perilesionais na ecoendoscopia e em outros sem metástases à distância. Os critérios de exclusão foram TNE >2 cm; os com infiltração da muscular própria; com adenopatias perilesionais e metástases à distância. As técnicas utilizadas foram: ressecção com alça de polipectomia; mucosectomia com injeção de solução salina; e mucosectomia após a ligadura com banda elástica. O estudo anatomopatológico dos espécimes incluiu avaliação das margens e imunoistoquímica (cromogranina, sinaptofisina e Ki 67) para caracterizar o tumor. O seguimento foi feito com 1, 6 e 12 meses.

**Resultados:**

Ressecções com alça de polipectomia foram realizadas em 15 pacientes; mucosectomia em cinco; mucosectomia e ligadura com banda elástica em três e os quatro restantes foram encaminhados para cirurgia. O anatomopatológico dos espécimes e as análises imunoistoquímicas mostraram cromogranina e sinaptofisina positivas, enquanto que o Ki 67 foi menor que 5% dentre todos os casos. O seguimento em médio prazo revelou três recidivas. A média de tamanho dos tumores no estômago foi de 7,6 mm e no duodeno 7,2 mm. As lesões bem demarcadas, hipoecóicas, homogêneas ocorreram em 75%; da camada mucosa em 80%; e da mucosa profunda e submucosa em 70%.

**Conclusões:**

A ecoendoscopia mostrou ser bom método para o estudo de lesões subepiteliais podendo identificar a camada acometida pela neoplasia, grau de invasão, ecogeneicidade, heterogeneidade, tamanho da lesão e acometimento linfonodal perilesional e melhor indicar a opção de tratamento.

## Graphical Abstract

[Fig f1as]

**Figure f1as:**
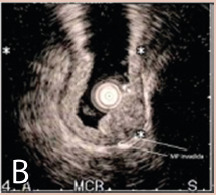
Tumor neuroendócrino gástrico mostrando invasão da camada muscular?

## INTRODUCTION

The non-functioning neuroendocrine tumor (NET) is the most frequent of all neuroendocrine tumors of the digestive system (73.7%) and occurs in the stomach/duodenum in 25%, in the rectum in 14%, appendix in 12% and pancreas in lower frequency^19^ ^,^ ^24^ ^,^ ^34^ ^,^ ^35^. They are being more diagnosed and American epidemiological surveillance data have shown that in the past 35 years their number in the small intestine has increased by about 300-500%^17^ ^,^ ^27^.

Gastric NET (NETg) type I tends to be benign, with a low risk of progression or metastasis^27^. Thus, the purpose of surveillance and treatment is a matter of debate. They make up 7% of all gastrointestinal NETs and 2% of all excised gastric polyps^3^ ^,^ ^4^ ^,^ ^27^. Those in the small intestine, especially those in the duodenum, are increasingly seen in early stages and are easily treated (with a diameter ≤10 mm) ^5^ ^,^ ^15^ ^,^ ^16^ ^,^ ^32^. They are generally non-functioning and found during upper digestive endoscopy, which is being performed for other reasons^9^ ^,^ ^11^ ^,^ ^18^. In case he has hormonal hypersecretion, the situation is different, more delicate and rare. Functional duodenal NETs (NEDs) usually metastasize at the time of diagnosis^7^ ^,^ ^8^ ^,^ ^13^ ^,^ ^25^ ^,^ ^26^. Probably NETg and NETd have been “overtreated” in the recent past, and as such, there is a current trend in directing more conservative treatments such as polypectomies and/or mucosectomies, in addition to endoscopic monitoring and surveillance. NETs <1 cm are resected by endoscopy, with endoscopic follow-up every six or 12 months. Many studies have shown that the successful removal of small NETg with mucosectomy does not have a frequent recurrence in long-term follow-up^10^ ^,^ ^26^ ^,^ ^28^ ^,^ ^31^.

Endoscopic resection must remove the tumor completely (R0 resection)^22^ ^,^ ^29^. To date, no recurrence has been observed after polypectomy/mucosectomy that affects the prognosis^20^. Echoendoscopy (EUS) has been increasingly used to assess the invasion of these tumors and to identify the presence of lymphatic metastases, in addition to determining the appropriate stage of the lesion^6^ ^,^ ^14^ ^,^ ^21^. Few studies assess its role with the intention of determining which are the best candidates for endoscopic resection^1^ ^,^ ^2^ ^,^ ^23^ ^,^ ^30^ ^,^ ^33^.

The objective of this study was to determine the effectiveness of EUS in staging subepithelial lesions identified by endoscopy in order to indicate the better form of treatment, endoscopic and/or surgical, and to evaluate the results of endoscopic removal in a medium-term follow-up.

## METHODS

This study was approved by the Ethics and Research Committee of Evangelical Faculty of Paraná, Curitiba, PR, Brazil, and all patients were previously informed about it and signed the informed consent used by the Endoscopy Department of 9 de Julho Hospital, São Paulo, SP, Brazil and the Section of Endoscopy of Hospital das Clínicas, Faculty of Medicine of Ribeirão Preto, University of São Paulo, Ribeirão Preto, SP, Brazil.

Twenty-seven patients with suspected NETs were treated in the cited services and submitted to EUS for TN tumor, TN staging and evaluation of the possibility of endoscopic resection, immediately after. All had subepithelial lesions identified by upper gastrointestinal endoscopy and/or biopsy with NET and underwent radial, sectoral or miniprobes EUS in the frequencies of 5.0, 7.5, 10 and 12 MHz. The examinations were performed with deep sedation using propofol with individual doses for each patient at the discretion of the anesthesiologist.

The EUS studied parameters were: size, layer of origin, depth of involvement (uT1=mucosa, uT1=submucosa, uT2=own muscle and uT3=serous affected) and perilesional adenopathies.

Those who met the following criteria were included for endoscopic resection: 1) high surgical risk; 2) NET <2 cm; 3) absence of impairment of the muscle itself; and 4) absence of perilesional adenopathies on the examination of EUS and ultrasound, tomography and resonance without distant metastases. NETs >2 cm were excluded.

The therapeutic endoscopy techniques were: polypectomy loop; mucosectomy with saline injection; and mucosectomy after ligation with an elastic band. In addition, anatomopathological studies were carried out, including evaluation of the margins, and immunohistochemistry with the removed part tested by chromogranin, synaptophysin and Ki 67.

The follow-up of the patients was obtained with imaging exams. Magnetic resonance imaging, computed tomography, digestive endoscopy and EUS at 1, 6 and 12 months were used. 

## RESULTS

**FIGURE 1:**
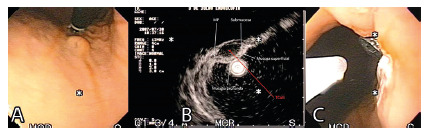
Patient with TNEg: A) endoscopic view; B) echoendoscopic view with the free muscle layer; C) after endoscopic resection

**FIGURE 2:**
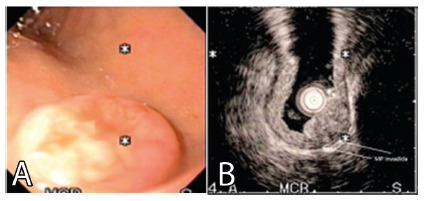
NETg patient referred for surgery: A) endoscopic view of the pylorus; B) EUS vision with muscle layer invasion?

The demographic characteristics of the 27 patients can be seen in Table 1. There were 16 men and 11 women with an average age of 59.4 years (34-78). Sixteen had NETg (Figures 1 and 2), two at the fundus, three in the proximal and middle body, 11 in the distal body. Eleven were NETd, nine in the first and 20 in the second duodenal portion. In this series, endoscopic biopsy diagnosed NET in 26/27 patients (96.2%). The finding of NET was incidental in 89% (n=24) and in 11% (n=3) carcinoid syndrome had been diagnosed only clinically, before endoscopy. The size of the tumors was assessed during this examination, and divided into two groups: less than or equal to 10 mm (52%) and 11-19 mm (48%).

**TABLE 1:** Demographic characteristics and variables evaluated (n=27)

Variables	Number of patients (%)
Patients	27
Genre	
Male	16 (59.3)
Female	11 (40.7)
Resected NET	23 (85.1%)
Number of procedures	29
Patients with multifocal NET	5 (18.5%)
Associated conditions	
Atrophic gastritis, type 1	4 (14.8)
Carcinoid syndrome	3 (11.1)
Location	
Stomach	16 (59.2)
Distal body	11
Proximal/midle body	3
Fundus	2
Duodenum	11 (40.8)
First portion	9
Second portion	2
Size	
<10 mm	14 (52)
11-19 mm	13 (48)
Resection technique	
Conventional technique - polypectomy loop	15 (55)
Mucosectomy with elevation (injection) of the submucosa	5 (34)
Mucosectomy after ligation with elastic band	3 (11)
Complete resection (free margins)	23/29 (79.3)
Complications	
Relapse	3 (11)
Abdominal pain	1 (3.7)
Duodenal perforation	1* (3.7)

* Patient died after several surgical procedures

Twenty-three patients (85%) underwent endoscopic resection and 29 NETs were resected. We opted for the conventional technique with polypectomy loop in 15, mucosectomy with injection of saline in five and mucosectomy after ligation with an elastic band in three patients. The anatomopathological study included a detailed evaluation of the margins and immunohistochemistry was performed with chromogranin, synaptophysin and Ki-67. Complete resection with free margins was possible in 23 of the 27 patients (79.3%). In addition, synaptophysin and chromogranin were strongly impregnated in the cytoplasm of the studied cells, characterizing the diagnosis of NET in the removed lesions. Ki-67, a nuclear marker of cell proliferation, showed low expression, being less than 5% in all removed NETs. As complications, a patient with abdominal pain and another duodenal perforation was obtained, being referred for surgical treatment. Three had tumor recurrence.

The parameters evaluated by the EUS were well-demarcated injuries (75%); hypoechoic, homogeneous, belonging to the mucous layer (80%); and deep mucosa of submucosal location (70%). Using the three parameters for the NET diagnosis in 27 patients a positive predictive value of 0.62 and a negative predictive value of 0.83 were obtained, with accuracy of 0.71. However, most of the false diagnosed lesions were located in the antrum (67%) and in the second portion of the duodenum (73%). EUS revealed that 22/27 NETs affected the superficial and deep mucosa; 4/27 (14.8%) the muscle itself and 1/27 (3.7%) the submucosa.

## DISCUSSION

NETs are rare and most are less than 10 mm in size, have a well-defined margin and are hypoechoic in nature; they are located in the deep mucous and submucous layers. The association of endoscopic findings (location, roughness, hardening), as well as the characteristics detected by EUS (echogenicity, heterogeneity and depth) are reasonable predictive factors for the differential diagnosis of gastric and duodenal subepithelial and polypoid lesions.

Previously, most NETs were treated by total gastrectomy, similar to adenocarcinoma^1^ ^,^ ^14^. In the last decades, NETg has been diagnosed early, and some have been treated by endoscopic resection (polypectomy/mucosectomy)^1^. Endoscopic resection techniques are now considered a viable option for the treatment of early gastric cancer, and their indications have been expanded^23^.

The use of EUS before treatment is increasingly recommended to assess the depth of tumor invasion, especially in cases of NET. On the other hand, other studies have shown that it may not be the ideal imaging modality for the NET diagnosis^33^. However, it is useful, as it offers additional preoperative information on depth, which is a very important factor in determining surgical resection instead of endoscopic resection, thus avoiding adverse events. EUS is quite accurate in differentiating the layers of the wall of the gastrointestinal tract and in defining the layer of origin of the tumor. Tumors can be found in any of the three layers and are slightly hypoechoic and homogeneous^30^. Thus, EUS decides whether a lesion can be safely resected by endoscopy or if surgical intervention is required^2^, a fact that occurred in this series.

Tumors with invasion confined to the submucosa can be treated by mucosectomy, while those with evidence of deeper invasion by surgical procedure.

The immunohistochemical study has proved to be of great value in the diagnostic process by means of neoplastic markers, such as synaptophysin, chromogranin and Ki 67. Synaptophysin, like chromogranin, has significant cytoplasmic impregnation in neoplastic cells, observed in the case series of this study. Ki-67, on the other hand, when it has high expression, is an important indicator of poor prognosis, which was not observed in this study.

In addition, after complete resection of the NETg, endoscopy with control biopsy should be routinely performed at six-month intervals, due to the risk of recurrence^2^.

Histological differentiation, location, type, biology, tumor stage and individual circumstances must be taken into account in the therapeutic planning of duodenal NETs. The treatment of non-functioning and well-differentiated, without risk factors for metastases limited to the mucosa/submucosa up to 10 mm in size and without vascular invasion, can be removed by endoscopy, as they have a low risk for the development of lymph node or distance metastases^2^ ^,^ ^12^.

## CONCLUSION

Echoendoscopy proved to be a good method for studying subepithelial lesions, being able to identify the layer affected by the neoplasm, degree of invasion, echogenicity, heterogeneity, size of the lesion and perilesional lymph node involvement, making endoscopic treatment safe and effective. With these indicators it allows to point out the best treatment, whether it is endoscopic or surgical.
